# Onset of nonlinearity in a stochastic model for auto-chemotactic advancing epithelia

**DOI:** 10.1038/srep33849

**Published:** 2016-09-27

**Authors:** Martine Ben Amar, Carlo Bianca

**Affiliations:** 1 Laboratoire de Physique Statistique, Ecole Normale Supérieure, PSL Research University; Sorbonne Universités UPMC Univ Paris 06; CNRS;, 24 rue Lhomond, 75005 Paris, France; 2 Institut Universitaire de Cancérologie, Faculté de médecine, Université Pierre et Marie Curie-Paris 6, 91 Bd de l’Hôpital, 75013 Paris, France

## Abstract

We investigate the role of auto-chemotaxis in the growth and motility of an epithelium advancing on a solid substrate. In this process, cells create their own chemoattractant allowing communications among neighbours, thus leading to a signaling pathway. As known, chemotaxis provokes the onset of cellular density gradients and spatial inhomogeneities mostly at the front, a phenomenon able to predict some features revealed in *in vitro* experiments. A continuous model is proposed where the coupling between the cellular proliferation, the friction on the substrate and chemotaxis is investigated. According to our results, the friction and proliferation stabilize the front whereas auto-chemotaxis is a factor of destabilization. This antagonist role induces a fingering pattern with a selected wavenumber *k*_0_. However, in the planar front case, the translational invariance of the experimental set-up gives also a mode at *k* = 0 and the coupling between these two modes in the nonlinear regime is responsible for the onset of a Hopf-bifurcation. The time-dependent oscillations of patterns observed experimentally can be predicted simply in this continuous non-linear approach. Finally the effects of noise are also investigated below the instability threshold.

Recently moving epithelium on a substrate has motivated many experimental^1^,^2^ and numerical works^2^,^3^ with the main aim to understand the collective migration of cell sheets occurring during development, inflammation, wound-healing^4^,^5^,^6^,^7^ or tumour metastasis^8^,^9^. Consideration of the movement of several thousand cells in a tissue of constant thickness can explain the mechanism of cell communication and migration. Intercellular interactions and substrate adhesion^10^, i.e mechanical forces, together with sensitivity to biochemical signals^11^ are responsible for these phenomena. One may argue that the importance of these morphogenetic signals depends on the experimental context, some cells being more chemotactic than others. However, in physiological conditions, chemotaxis cannot be ignored, e.g. as in the wound-healing process: Just after injury^4^, a battery of morphogens originating from the immune system organizes the migration of several connected cell layers of the basal epithelium. In particular, it has been recognized that cells can produce their own chemoattractant, as melanocytic cells. For these cancerous cells, it is a way to escape the tumour and undergo metastasis^9^. As shown in Fig. 1, in the circular geometry^12^, *in vitro* advancing epithelium of melanocytic cells exhibits simultaneously a noisy front and a diffuse interface, which strongly suggests the local production of chemo-attractants.

This paper is concerned with the so-called auto-chemotaxis process where cells themselves produce their own chemo-attractants. Auto-chemotaxis can be considered as a mechanism for neighbour cell communication, which by transforming biochemical signaling into mechanical stresses induce collective migration^13^. This process has also been investigated in the dynamical aggregation of dense micro-swimmer colonies^14^. It is worth stressing that, even in extremely well controlled experiments, it appears difficult to distinguish between chemotaxis and mechanotaxis^10^,^13^,^15^ (including all forces and stresses that a cell can feel in a moving epithelium). In recent theoretical approaches for *in vitro* advancing epithelia, chemotaxis has not been considered and discrete cellular models with short-range interactions, treated with simulations eventually including noise, are preferred^2^,^16^. However, here the choice is made of a continuous model to explain experimental features, such as the origin of waves occurring during tissue expansion^16^.

Pattern formation has a rather long history in physics, in diffusion of growing crystals^17^,^18^, viscous fingering in fluids^19^,^20^, electrostatics^21^,^22^ and soft matter^23^. Theoretical methods have been developed to understand the front dynamics originating from the interplay between interfacial and bulk processes. If continuous models have been proposed in the past for advancing epithelia^3^,^24^,^25^, simulations are preferred in most cases, although nonlinear analysis of pattern formation can help in the understanding of the collective motion of cells when the experimental size is much larger than the cell size. This paper aims at explaining the pattern formation observed in recent experiments^12^,^15^,^26^, by means of the definition of a continuous model of auto-chemotactic migration, including also cell mechanics and cell proliferation. We show that the nonlinearity coupled to the inherent stochasticity of any biochemical signaling induces pattern formation below the instability threshold and we evaluate the velocity structure function which is a measurable parameter.

The paper is organized as follows: after a presentation of the system of study, a continuous theoretical model is presented and an advancing steady front is theoretically predicted. A stability study restricts the range of physical parameters allowing periodic patterns that are determined at the linear approximation. Then the stochasticity and the nonlinearities are introduced to predict the wavy dynamics observed in the quasi-infinite geometry.

## The model

### Objectives and Hypotheses

A theoretical model is proposed to investigate the dynamics of a cellular sheet, controlled by auto-chemotactic gradients and cellular proliferation. A front separates the experimental cells into two domains Ω_*e*_ (the epithelium) and Ω_*w*_ (the water and nutrients), as shown in Fig. 2. Physical quantities characterizing the front, such as velocity, stress repartition, stochastic and spatio-temporal patterns, will be predicted with a minimal set of parameters according to the following strategy: steady leading edge dynamics, stochastic and wavy edge, spatio-temporal front, (see the three schemes of Fig. 2). Among the three scenarios, from left to right, there is an increase of the chemotactic gradients which controls the evolution: first an advancing linear epithelium edge with constant velocity, second a wavy edge, also advancing with constant velocity, then above the stability limit, a spatio-temporal pattern.

The biological description is based on simple assumptions:

- The cellular proliferation occurs as soon as the level of tension inside the cells is higher than the homeostatic one, according to a mechano-sensitive growth process^7^,^12^,^27^,
- On the substrate the motility law is dominated by friction,
- The cells communicate via chemical signals giving rise to auto-chemotaxis.

**Figure 1:**
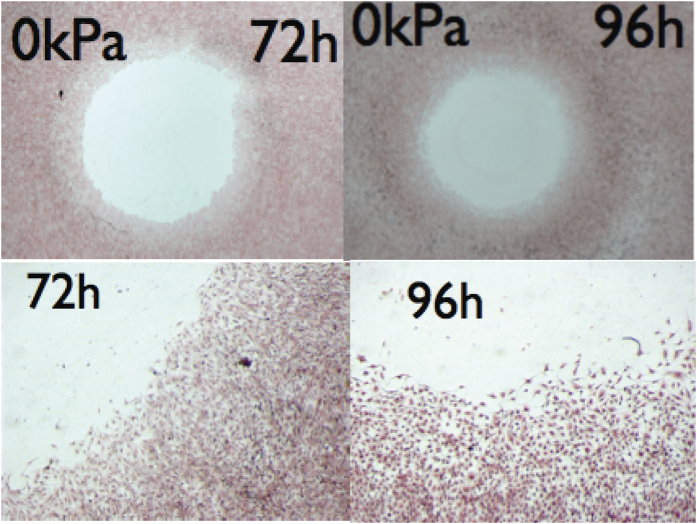
Human melanocytic cells (primary tumour) were plated on 33 cm^2^ Petri dish with at the center a disk (32 mm^2^) at confluence. The day after, the disk was removed and cells were allowed to keep migrating and dividing, before they were fixed with 3.7% paraformaldeide and stained with hematoxillin/eosin solution, at different times. On top, typical experiment, at two different times, on bottom, details from the same images with X5 magnification, compared to the top (Leica MZFLIII mounted with a camera Leica DFC320). Observe the diffuse front and an increase of noise after 4 days. From publication^12^ with permission.

Auto-chemotaxis and proliferation are two factors of migration that occur simultaneously. In order to limit the number of independent parameters in our analysis, we assume the same diffusion coefficient *D*_*e*_ for the morphogens inside the cellular domain Ω_*e*_ and in the water bath Ω_*w*_. In Ω_*e*_, the morphogens diffuse but are also captured by the cells and disappear with a characteristic uptake *τ*_*e*_. The cells of the interface produce more morphogens than inside the epithelium where these chemicals are absorbed, so only a small number of rows are responsible for the morphogen production. Notice however that cells away from the front also produce morphogens but in less quantity that the ones at the frontier. In addition, it is known that epithelia have a constant thickness, except at the boundary, where a thinning can be observed as in Fig. 1 and reported^12^,^28^. Based on these biological informations and hypotheses, the main equations follow.

### The main equations

The morphogen concentration satisfies the diffusion-consumption equation in the tissue and the diffusion equation in water:


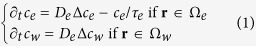


Let us define dimensionless physical quantities as capital letters and choose *τ*_*e*_ as time unit, 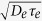 as length unit and as concentration unit, the averaged value at the front, *c*_*i*_ = (*c*_*e*_ + *c*_*w*_)/2. Then, the equation (1) are transformed into:


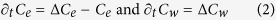


At the front of normal 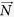, oriented towards Ω_*w*_, the jump conditions for both the concentration field and its normal gradient read:


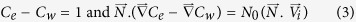


The first relation of equation (3) gives the normalized jump in the morphogen concentration while *N*_0_ indicates the rate of morphogen production at the border, responsible for the flux discontinuity. These relations indicate a discontinuity due to the localization of the morphogen sources, attached to the front and being transported with it. These relationships are similar to the boundary conditions of the so-called one sided-model of solidification for binary mixtures^17^ and they have been mathematically established^29^ and derived in chemotaxis^30^. In both domains, far from the interface, *C*_*e*_ = *C*_*w*_ = 0. When the friction on the substrate^31^ dominates the sheet motility, the cell velocity follows a Darcy’s law: 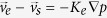 where *K*_*e*_ is the mobility coefficient. 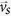 is usually neglected since most of the experimental set-ups are at rest in the laboratory frame. However, this formulation makes more explicit the Galilean invariance of the Darcy’s law and underlines the fact that friction results from velocity differences^32^,^33^,^34^. In the laboratory frame, we transform Darcy’s law into 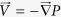, once the unit of pressure is chosen as *P*_0_ = *D*_*e*_/*K*_*e*_. This law can eventually be modified into a power law model used in ref. 30 where the mobility coefficient *K*_*e*_ is itself a function of the pressure gradient. The cells proliferate with a rate *K*_*p*_ as soon as the pressure is below the homeostatic pressure *P*_*h*_^12^,^27^, (chosen as our pressure of reference) which leads to the following mass-flux equation:


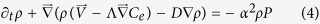


where the chemotactic migration constant is introduced: Λ = *λ*_0_*c*_*i*_/*D*_*e*_, suppress the cell diffusion coefficient: *D* and the coefficient of proliferation: *α*^2^ = *K*_*p*_*D*_*e*_/*K*_*e*_. Equation (4) superposes the convective flux to the diffusive and chemotactic fluxes^35^. For an epithelium of constant thickness (not true at the interface), *ρ* is a constant and the model assumes a priori cohesiveness and excluded volume interactions. Contrary to discrete simulations, these two constraints are automatically verified. Moreover, far from the front, we get for the pressure the simplified equation: Δ(*P* + Λ*C*_*e*_) − *α*^2^*P* = 0. However chemotaxis does not allow a constant thickness at the front^30^ and depletion of density is experimentally observed^12^,^28^ (see also Fig. 1). This density depletion is necessary to ensure the mechanical equilibrium of the interface as it has been demonstrated^30^ in details. Indeed, an abrupt front under chemotaxis does not allow to maintain simultaneously two constraints: on the one hand, the equality of cellular and interface normal velocities, and on the other hand, the cancellation of chemical flux. The cellular density variation and diffusion, inside the boundary layer, give the necessary degrees of freedom allowing this subtile equilibrium by imposing: 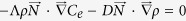. If the size of the transition zone remains small, the mechanical equilibrium of the front fixes the boundary condition for the pressure:


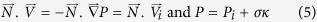


where 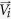 is the interface velocity. The interface pressure *P*_*i*_ is the pressure in the water bath, relative to the homeostatic pressure, the curvature *κ* is chosen positive for convex interface and *σ* represents the dimensionless capillary parameter 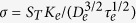, proportional to the surface tension *S*_*T*_. Before going further, it is worth noticing that, for an epithelium advancing in one direction in an infinite experimental set-up, the Galilean invariance is verified for this set of equations and in particular for equation (4). Indeed a change of Galilean frame induces a substrate velocity 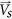 and a convection term 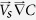 must be added into the left-hand side of equations (2). Also a modification of 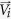 into 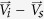 is required into the boundary condition (3). Because it makes more complex the writing of equations, the choice of the laboratory frame is often preferred as in equation (5), destroying apparently (but only apparently) this fundamental symmetry. Changing the velocity 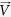 into 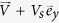 and transforming *ρ* and *C* into *ρ*(*X*, *Y* − *V*_*s*_*t*, *t*) and *C*(*X*, *Y* − *V*_*s*_*t*), *t*) restore the initial equations (2) and (4) by a simple change of coordinates, confirming the Galilean invariance of the model.

### Free boundary problem and stability analysis

**Figure 2:**
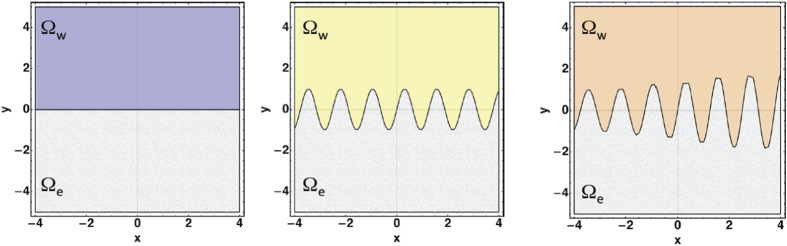
The three steps of leading edge evolution as the auto-chemotactif gradients increase. In grey, the cell sheet (Ω_*e*_), in colours, the bath of water and nutrients (Ω_*w*_). From left to right, a steady front with constant velocity, a wavy front at threshold of stability, then the spatio-dynamical pattern.

The usual step for the analysis of such free-boundary problem consists in the search of a simple solution moving with a constant velocity and the study of its stability. For a steady traveling front, Fig. 2, left panel, moving with the velocity *U* along the *y* axis, both concentrations *C* vary exponentially in the co-moving frame of velocity *U*. Elementary algebra gives for the epithelium:


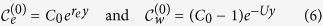


where 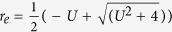 and *C*_0_ = *r*_*e*_*U*(1 + *N*_0_). The pressure results from proliferation and chemotaxis:


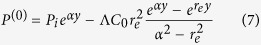


which gives the interface velocity through an implicit relationship, function of the chemotactic forcing Λ and the proliferation rate *α*:


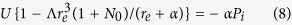


**Figure 3:**
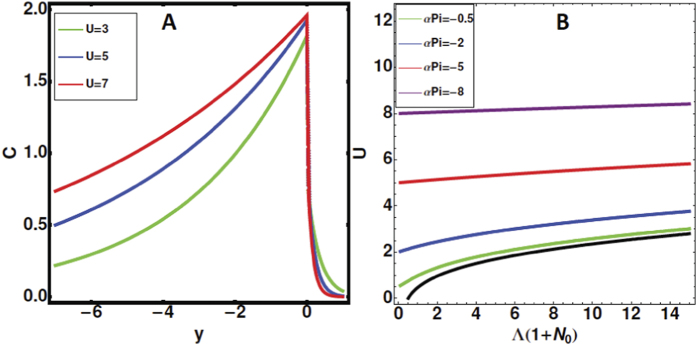
Left panel (A): Density profile of the morphogens as a function of the distance from the front position *y* = 0, in the experimental cell. Velocities are increasing from 3 to 7 while setting *N*_0_ = 1. Right panel (B): The front velocity as a function of combined chemotactic parameters, when varying the interface pressure and setting *α* = 1 (see equation (8)).

Figure 3 summarizes the results for a particular choice of the parameters. The density (Fig. 3A) and pressure fields vary exponentially with the distance from the front, only the morphogen concentration is given, having its maximum at the front. Notice that fast fronts maintain the morphogen concentration on large distances. As *U* decreases, the morphogen concentration is more localized around the leading edge but for higher velocities (so higher cellular proliferation), one may observe a spreading of the morphogen concentration. In the water, the decrease is abrupt, but this event does not affect the cells. Figure 3B shows the front velocity as a function of the combined parameters Λ(1 + *N*_0_) and *αP*_*i*_. From equation (8), we recover the two limiting cases: A front driven only by chemotaxis without proliferation, having velocity: 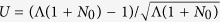) and a proliferative front with velocity: *U* = −*αP*_*i*_. Let us consider now the stability of such fronts. The stability analysis of such fronts is performed by introducing a sinusoidal perturbation induced by a wavy frontier of the cellular domain: *ζ* = *Ut* + *εe*^*ikx*^*e*^Ω*t*^ (in analogy with the Fig. 2, middle panel) and expanding all fields according to:


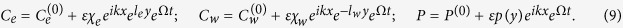


**Figure 4:**
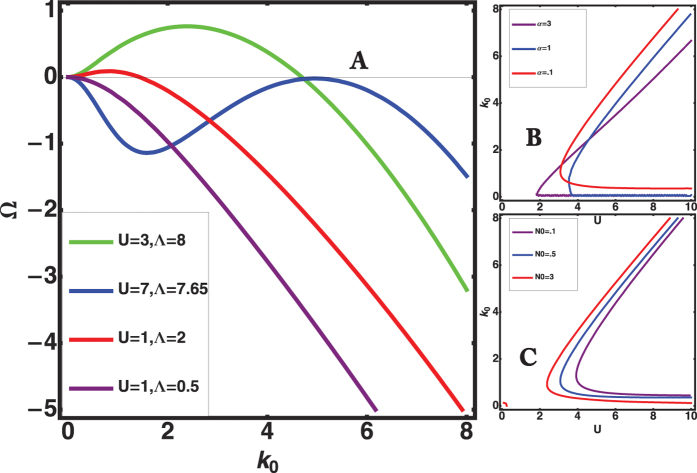
(**A**) The growth rate Ω versus the wavenumber *k*_0_ when *U* and Λ vary at fixed values: *α* = *N*_0_ = 1 and *σ* = 10^−3^. Note that the chemotactic flux (proportional to Λ) is destabilizing contrary to the advective one, proportional to *U*. (**B**) Selected wavenumber *k*_0_ versus the front velocity *U* for *σ* = 10^−3^, *N*_0_ = 1 and *α* is varied from 3 to 0.1. (**C**): *α* is fixed to 0.5, *N*_0_ varies from 3 to 0.1.

At linear order in *ε*, we have:


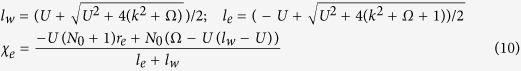


where the growth rate Ω reads:


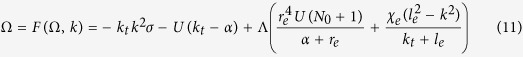


with 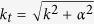. The dispersion relation, equation (11), which predicts stable steady fronts for Ω < 0, is not easy to analyze owing to the implicit relation for the growth rate Ω, *l*_*w*_, *l*_*e*_ and *χ*_*e*_ also being functions of Ω. However, one easily notices that, proliferation alone (without chemotaxis) makes the front stable, Ω being negative, and the increase of the parameter Λ may induce an instability. Setting *N*_0_ = *α* = 1 and *σ* = 10^−3^, numerical investigations show the existence of stable patterns when Ω remains negative for all *k* values (purple curve of Fig. 4A). They also detect fully unstable patterns, out of reach of the present analysis (green and red curves where Ω > 0 for a finite interval of *k* values). For these two cases, we go directly from a stable planar front to a developed instability without periodic wavy patterns (a situation commonly encountered in diffusive instabilities such as dendritic growth^17^). Finally, from Fig. 4A, we can predict steady periodic patterns of finite amplitude (blue curve) since the spectrum exhibits the simultaneous cancellation of Ω and *d*Ω/*dk*. This requirement of double cancellation is mandatory for observation of steady undulating patterns. From this analysis, we deduce that a steady wavy-front with prescribed wavelength *l*_0_ and wavenumber *k*_0_ can be observed, travelling with a constant velocity *U* above a critical value of Λ called Λ_0_. Fixing the front velocity and the parameters *σ*, *N*_0_, *α*, as shown in Fig. 4B, or Fig. 4C, a pair of solutions for *k*_0_ can be numerically found so a pair of Λ values. This pattern at *k*_0_ can explain the fingering pattern observed in experiments^1^,^2^,^12^. As for the mode observed at *k* = 0, it results from symmetries as the translational and Galilean invariances (called Goldstone mode). It will play an important role for the nonlinear analysis.

This linear analysis can predict a fingering instability of the leading edge. However, at this stage of linear order, it is not sufficient because these two modes *k* = 0 and *k* = *k*_0_ compete, in the nonlinear regime, thus requiring a careful nonlinear study. Moreover, the relative disorder observed in moving epithelia, leading to fingering and noisy fronts, also suggests that a stochastic study may be helpful to understand the observed patterns.

### Nonlinearity and Stochasticity

**Figure 5:**
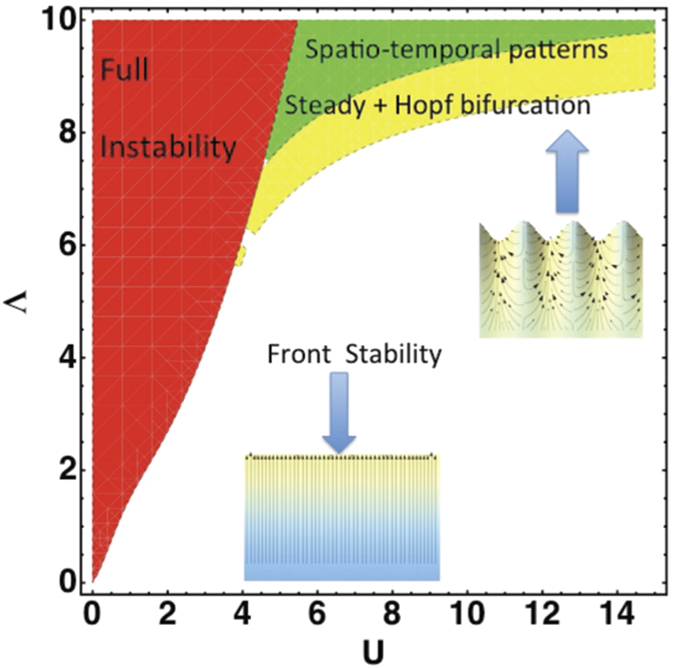
Phase-diagram Λ versus the averaged front velocity *U* for the parameter values corresponding to Fig. 4, on left: *α* = *N*_0_ = 1, *σ* = 10^−3^. The fully stable domain is in white (without noise), in green the estimated weakly nonlinear domains with steady advancing periodic patterns (represented below) and Hopf bifurcation, in yellow, the domain of spatio-temporal pattern, not treated here as the fully unstable domain represented in red. In the inset, schematic representation of the steady pattern, the pressure at the interface is fixed to *P*_*i*_ = −6.76, giving *U* = 7 and *k*_0_ = 5.

The previous analysis was restricted to weak perturbations of the planar front represented in Fig. 2, left panel leading to oscillations of tiny amplitude represented in Fig. 2, middle panel when the parameter Λ is close to Λ_0_. We consider now the disorder which always exits in an experiment with cells, especially when they are cancerous. If many theoretical works have been devoted to patterns with noise in experiments of hydrodynamic such as the Rayleigh-Benard^36^,^37^, it is less true for free boundary problems, except for the side-branching instability in dendritic growth^38^,^39^,^40^. The noise originating from the biochemical signals will modify equation (3) and equation (5) as explained in Section **Stochastic fronts**. However, the analysis of stochasticity will be made easier if we can establish an effective weakly nonlinear equation, close to the threshold Λ_0_ and wavenumber *k*_0_. Indeed, when Λ steps across the threshold Λ_0_, one can approximate locally the dispersion relation, equation (11), by:


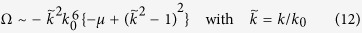


where *μ* represents the gap from threshold: 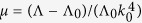. This suggests a nonlinear equation for the interface velocity deviation *u* (such as *V* = *U*_0_ + *u*) which recovers the dispersion relation at threshold (equation (12)):


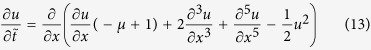


where 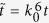. How to select the nonlinearity in equation (13)? Above threshold, the first nonlinearity is −*β*^2^*u*^2^ where *β* is a numerical value and the negative sign indicates a saturation of the velocity amplitude. There is no symmetry *u*− > → −*u*, since the velocity correction *u* cannot behave symmetrically in the front direction (upstream) and in the opposite direction (downstream). Defining 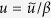 eliminates the unknown constant *β* from the equation for 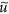 and we recover equation(13). However a more fancy way consists in applying the Galilean invariance symmetry (1/2∂*u*^2^/∂*x*): *x* → *x* − *vt* and *u* → *u* + *v*. Such consideration allows to avoid the very tedious nonlinear analysis from the free-boundary equations. This quadratic nonlinearity also imposes the scaling of the parameters: Choosing |*μ*| of order *ε*^2^, from the dispersion relation, we deduce that the lengths scale as 1/*ε* while, from the right-hand-side of equation (13), the velocity *u* scales also as 1/*ε*. The left-hand-side gives us the time-scale as 1/*ε*^2^. As shown in Fig. 1, chemotaxis, which is responsible for diffuse fronts, increases stochasticity and the disorder. Restricting to the biochemical noise in our model, stochasticity enters at the level of the boundary conditions: The jump condition for both concentration and gradient of concentration and the chemotactic migration constant Λ. Stochasticity from the pressure and the friction of the cells at the substrate are neglected. We are then face simultaneously to a multiplicative noise *η*_1_, indicating a noisy threshold for bifurcation and an additive noise *η*_2_ arising from all other sources. Both sources of noise are chosen Gaussian in space, white in time and are not correlated so 〈*η*_*i*_(*x*, *t*)*η*_*j*_(*x*′, *t*′)〉 = *C*_*j*_(*x*)*δ*_*ij*_*δ*(*t* − *t*′)*δ*(*x* − *x*′). Restricting on the vicinity of the interface, close but below the bifurcation threshold *μ* < 0, (which corresponds to the white zone of parameters in Fig. 5), we transform equation (13) into a stochastic equation adding *η*_1_ and *η*_2_. It reads:


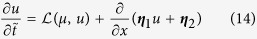


where 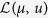 is the differential operator, right-hand-side of equation (13). The correspondence between the biochemical noise and ***η***_1_ and ***η***_2_ is established in Section **Stochastic fronts**. Below the instability threshold, the velocity *u* has only a stochastic component and neglecting the non-linearity and the multiplicative noise contributions, the velocity structure function reads 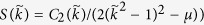, (see Section **Stochastic fronts**), a result similar to the linearized Swift-Hohenberg equation derived in refs 36 and 41, indicating that periodic patterns can be observed below the threshold *μ* = 0. The effect of the multiplicative noise can be approximated by using Novikov’s theorem^37^ leading to a new contribution proportional to 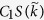 which modifies the threshold *μ* becoming *μ* + *C*_1_. It contributes to a possible jump into the unstable domain even if the parameter *μ* is negative. Finally the quadratic nonlinearities do not contribute, in opposition to the Swift-Hohenberg equation^41^. This analysis allows to conclude that below the threshold of instability of the planar front, it is possible to observe a wavy pattern induced by a small level of noise. However, above the threshold, noise may play a role only when it competes with the nonlinear effects so very close to the onset. If its level is too low, nonlinearities dominate.

### Nonlinearity and the Turing-Hopf bifurcation

For positive *μ* values, the velocity *u* results from the competition between both modes at 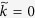 and at 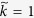, respectively, which are coupled by the nonlinear terms. This competition suggests the following decomposition 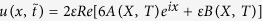, *B* representing a small change in the front velocity, *X* and *T* being rescaled variables: *X* = *εx* and 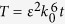. A classical multi-scale analysis^41^,^42^ gives us the coupled amplitude equations for both modes *A* and *B*:


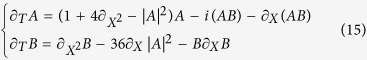


**Figure 6:**
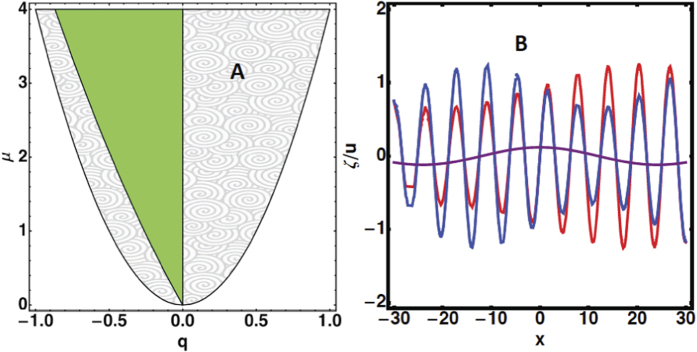
Left panel (A): Domains of spatial wavelengths *q* = (*k* − *k*_0_)/*k*_0_ in the nonlinear regime defined by *μ* = (Λ − Λ_0_)/Λ_0_. In grey, unstable modes, in green stable modes. The left-hand frontier between grey and green domains corresponds to the Hopf bifurcation. Right panel (B): Schematic representation of the interface profile *ζ* and the average front velocity, for *μ* = 0.1. The interface oscillates in time between the blue and red curves, the curve in purple indicates the mean velocity.

The last terms of the first equation (15) represent the advection of the pattern, 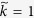, by the change in the front velocity *B*. We also notice that *B* has only a nonzero value for inhomogeneous values of the amplitudes *A* and *B* (see Section **Hopf-Bifurcation**). Equations (15) are similar to those established previously^43^,^44^ and such coupling has been called Hopf-Turing bifurcation^42^. Exact solutions for *A* and *B* in equation (15) can be easily found: *A* = *A*_0_*e*^*iqX*^ with amplitude: 
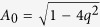
 and *B* = 0 (see the domain in green in Fig. 6A). A stability analysis of these solutions is necessary knowing that their domain of existence is constrained by the inequality *q*^2^ < 1/4. Once perturbed according to *A* = (*A*_0_ + *r*)*e*^*iqX*+*ϕ*^ and *B* =*ψ*, where *r*, *ϕ* and *ψ* a*r*e small real functions of wavenumber *p* and growth rate *η*, an algebraic equation is established (see Section **Hopf-Bifurcation**) for the growth rate *η* giving modes growing exponentially in time for *q* > 0, a range of stable modes for negative *q* values, bounded by a Hopf bifurcation for *q* = *q*_*h*_ (see the schematic representation of the analysis in Fig. 6A where unstable modes are in the grey domain, stable modes are in the green domain and the Hopf-Turing modes are at the frontier on left). For 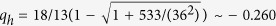, waves appear and superpose to the static spatial mode *q*_*h*_ affecting simultaneously the spatially periodic oscillation *A* and the average velocity *B* of the front. The interface oscillates between the blue and red curves of Fig. 6B, but also the average velocity of the front (represented in purple). For a number *p* of the wave, the frequency for the time-dependence is equal to *ω*_*h*_*p* with 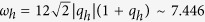.

The nonlinear analysis proves that there exists a band of stationary spatial oscillations in the neighbourhood of 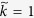, with amplitude 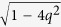 and wavenumber 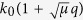, where *q*_*h*_ < *q* < 0. At the frontier, *q* = *q*_*h*_, the frequency of the time dependent oscillations becomes 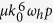. Also the averaged front velocity oscillates with the same frequency 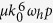. Let us evaluate an order of magnitude for the oscillation period *T*_*h*_ and compare our results with measurements performed in ref. 16. From the evaluation of the ratio between the amplitude of the fingering and the spatial wavelength^16^, 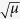 is estimated as 0.1/12 (the factor 12 comes from the definition of *A*). The wave-number *p* can be deduced from the measured strain-rate 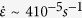, which identifies to 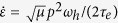. As shown in ref. 45, *τ*_*e*_ varies between 45 minutes to two hours while *D*_*e*_ = 0.3(*μ*m)^2^/*s*. Taking the lowest estimation for *τ*_*e*_, we find *p* ~ 0.15 which gives a period *T*_*h*_ = 400*s* for *k*_0_ ~ 7.2. From Fig. 4, on right, such *k*_0_ value indicates a velocity *U* ~ 10, giving a front velocity 10(*D*_*e*_*τ*_*e*_)^1/2^ ~ 0.1*μ*m/*s* while the wavelength is about 25*μ*m. These estimations are in good agreement with the experimental results^16^. A phase-diagram (Λ_0_, *U*) is presented in Fig. 5 for fixed values of *N*_0_ = *α* = 1 and *σ* = 10^−3^ which shows the domain of a planar front (with velocities oriented towards the leading edge), the homogeneous oscillatory patterns combined eventually, with a Hopf bifurcation (in yellow). We show also the domain of full instabilities with spatio-temporal dynamics (in green) and the fully developed instabilities (in red) which are not studied here.

The main conclusion of this analysis is that temporal global modes of oscillations is the result of the Galilean invariance of the experimental set-up, which occurs in nonlinear systems as soon as their dispersion relation presents a similar behaviour as Fig. 4.

## Discussion

Epithelial monolayer expansion is mostly regarded as the interplay between mechanical forces combined with stochasticity. The samples involved in many experiments^1^,^2^ have sizes of order 100*μ*m and perhaps cannot be compared with the example shown in Fig. 1 which is of order centimeters and lasts for several days. It is not an easy task to quantify precisely the magnitude of auto-chemotaxis and its contribution to motility. In addition, it probably depends on the nature of the cells. However it deserves to be studied as a possible mechanism for *in vitro* tissue motility. Our macroscopic model averages the mechanical forces acting at the cellular level which is justified by the coordination existing among cells in the bulk, far from the border. The model suggests a different scenario via the exchange of chemical factors made by the cells themselves and gives front undulations and front temporal oscillations. These waves result from the combined effects of the translational invariance (responsible for the mode *k* = 0), the existence of a spatial undulation (mode *k* = *k*_0_) detected by the linear stability analysis, equation (11), and the nonlinearities deduced from the Galilean invariance. It is different from other instabilities where waves appear at linear order via a complex growth rate Ω in the dispersion relation^46^. Although we cannot quantify the level of chemical signaling, any diffusive front is a strong indication of auto-chemotaxis^26^,^30^. The experiment shown in Fig. 1 does not respect the translational invariance required by the model but it shows a diffuse front. Once the initial geometry is modified to correspond to the free planar growth, some aspects of the theory can be verified since the model gives analytical predictions. Different parameters can vary such as the mobility coefficient *K*_*e*_ (by changing the substrate) or the proliferation rate (by adding a biocompatible polymer, dextran, to the solution). It will allow to explore the phase diagram shown in Fig. 6. It has been shown that dextran induces a controlled osmotic pressure^12^,^47^ so allows to vary the interface pressure *P*_*i*_. Among quantitative measurements, the velocity structure function, predicted here equation (21), is an accessible quantity in most of the experimental set-ups^1^,^2^. A more systematic study of the waves, detected above threshold in a specific domain of parameters, will be highly valuable.

## Methods

### Stochastic Fronts

Here, we rely the additive and multiplicative noise term ***η***_1_ and ***η***_2_ of equation (14) to the biochemical and physical sources of noise. Stochasticity has been introduced in nonlinear systems, near threshold of bifurcations via effective equations called stochastic normal forms. The most studied equation is probably the stochastic Swift-Hohenberg equation (for a review, see ref. 37). As for our front nonlinear equation (13), the main advantage is the simplicity of these approaches compared to the full hydrodynamic equations. Neglecting the nonlinearities, the Fourier transform of equation (14) gives


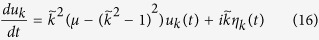


where, the noise correlation function, for a white noise in time but coloured in space ***η***_2_ is : 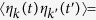

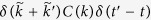
. We define 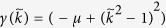, which is positive below the threshold of instability (*μ* being negative). The velocity correlation function, 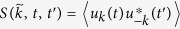, where u^*^ denotes the complex conjugate of *u*, is evaluated explicitly in the following. Indeed, from equation (16), we derive:


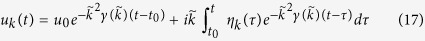


The long time behaviour, *t*_0_ → −∞, cancels the first term in the right-hand side of equation (17). The function 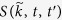 satisfies


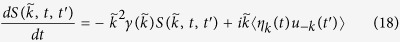


In order to evaluate the quantity 〈*η*_*k*_(*t*)*u*_−*k*_(*t*′)〉, from equation (17), we derive:


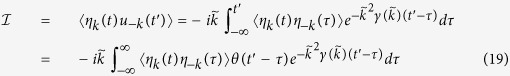


where *θ*(*X*) is the Heaviside step function, equal to 1 for *X* > 0, to 0 for *X* < 0, and to 1/2 for *X* = 0. At this step any coloured noise in time and space can be introduced as example a Gaussian distributed noise with zero mean. However, to simplify, we choose a noise term with zero mean, white in time. Accordingly, it reads:


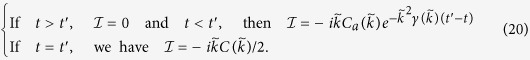


At long times, the structure function reads:


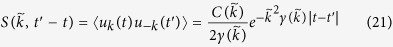


Close to the threshold, given by *μ* = 0, for negative values of *μ*, the only root of 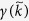 is 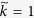, which increases the velocity structure function for this value. Therefore, at linear order, fingering with 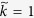 is selected by the noise below the hydrodynamic threshold. However, at threshold, the structure function diverges for 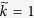 and nonlinearity cannot be discarded. Let us identify the origin of the multiplicative and additive noises for our specific free boundary problem.

#### Stochasticity and free boundary problems

The correspondence between the biochemical or biophysical noise and the stochastic variables introduced into equation (14) requires to come back to the free boundary problem, where the nature of the noise is well identified. The interplay between nonlinearities and stochasticity is a challenging theoretical problem for fully developed instabilities^38^,^39^,^40^ but becomes easier for patterns of finite amplitude like the one investigated here. However the origin of noise and its nature (additive or multiplicative) have to be elucidated. Patterns result from the interplay between fields, solutions of partial differential equations and initial boundary conditions. These problems give rise to strong nonlinear instabilities and noise is not always a pertinent parameter, except close the threshold of instability. Focussing on chemotaxis, the chemical production of morphogenetic gradient is surely a source of noise, however, the cellular activity such as mitosis is also a noisy mechanism. Here we restrict to noise arising from the morphogen production. The morphogens, occurring directly at the border, may affect more the fingering instabilities. Mathematically, morphogens enter into the chemotatic migration constant Λ and the two boundary conditions in equation (3). This leads to three stochastic variables that we consider uncorrelated.

#### The multiplicative noise

The multiplicative noise is easily identified. The phase diagram has demonstrated that a possible control parameter is the average front velocity *U*, equation (8), which is itself related to the chemotactic flux. In equation (8), stochasticity appears through the parameter *N*_0_ transformed into *N*_0_ + ***η***^(4)^, and the chemotactic migration constant Λ which becomes Λ + ***η***^(5)^. A straightforward calculation shows:


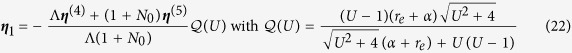


##### Numerical estimation

For our choice of parameters, *α* = *N*_0_ = 1, Λ = 7.65 and *U* = 7, ***η***_1_ can be estimated and gives ***η***_1_ = −(0.5***η***^(4)^ + 0.26***η***^(5)^) with 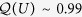.

#### The additive noise noise

Each boundary condition (equation (3) and equation (5)) is modified by a stochastic contribution ***η***:


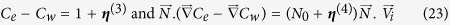


We will solve the free-boundary problem linearly, by using the Fourier transform for the three noise sources ***η***^(3)^, ***η***^(4)^ and ***η***^(5)^ as follows:


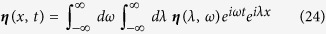


and for the interface:


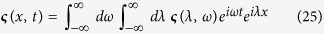


The calculation is made simpler if we do not consider the time variation of the noisy terms in both diffusion equations and we derive ***η***


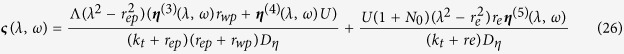


with 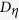



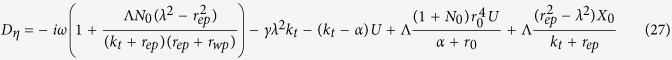


where 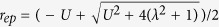, 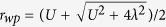 and *X*_0_ = (−*U*(*N*_0_ + 1)*r*_0_ − *N*_0_*U*(*r*_*wp*_ − *U*)/(*r*_*ep*_ + *r*_*wp*_). Using equation (11), we transform *D*_*η*_ as follows :


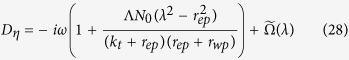


and we introduce


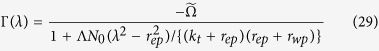


Remember that 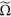 remains negative below threshold. Then, the noisy interface equation (26), now reads:


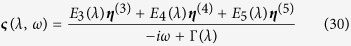


where *E*_3_, *E*_4_ and *E*_5_ are given by:


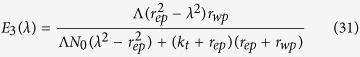



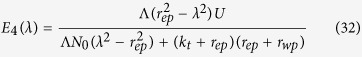



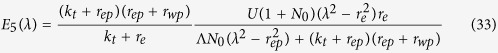


#### The Langevin equation for the front position

It is more easy to analyze the free-boundary problem with noise by writing the equivalent Langevin equation for equation (30):


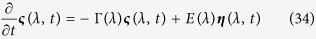


Since the three sources of noise in equation (30) are independent, we can treat each of them separately. Applying the time Fourier transform to equation (34), we recover the same structure as equation (30), which facilitates the identification of the noise sources occurring in the free boundary problem and in equation (14). Equation (34) can be integrated in time giving:


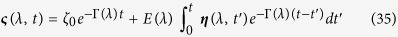


Since 〈***η***(*λ*, *t*)〉 = 0, then the averaged value of the front position in the moving frame vanishes with time: 〈*ζ*〉 = *ζ*_0_*e*^−Γ(*λ*)*t*^ whether the noise appears at time *t* = 0. Let us evaluate the auto-correlation function:


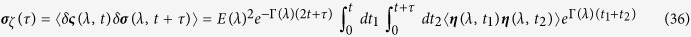


If we take a white noise in time but coloured in position, we have *C*(*τ*) = *C*_0_(*λ*)*δ*(*τ*) and then:


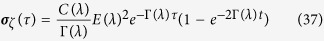


Clearly ***σ***_*ζ*_(*τ*) increases in the neighborhood of *λ* = *k*_0_ and the noise spatial frequency is selected even below the threshold. In the neighbourhood of *λ* = *k*_0_ a regular pattern induced by noise is expected. Not surprisingly, near *λ* ~ *k*_0_, the auto-correlation function of the position becomes linear in *t*. In addition the structure function, which corresponds to ***S***(*λ*) = ***σ***_*ζ*_(0) and *t* → ∞, can be evaluated in the vicinity of *λ* ~ *k*_0_. Indeed, once Γ(*λ*) is approximated by 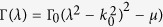, it reads:


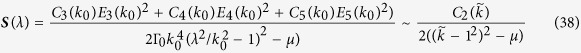


where *μ* is the negative gap from the hydrodynamic threshold of instability. The relationship between the noise introduced in the nonlinear equation (14) and the biochemical noise is now established. Obviously *η*_2_ turns out to be a nontrivial combination of the biochemical sources produced at the frontier.

### Hopf-Bifurcation

The non-linear front velocity equation (13) cannot be solved naively with a Fourier series expansion Σ_*m*_*a*_*m*_*e*^*imx*^, where *m* is integer. Indeed, because of the nonlinear term 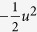, the related series will include secular terms which produce its divergence^48^. In order to eliminate these terms, one choice consists in considering the following decomposition^42^:


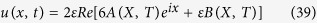


where *X* = *εx*, 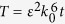, *μ* = *ε*^2^, and *μ* = *ε* = 0 at the instability threshold, which transforms the nonlinear equation (13) into the two following amplitude equations:


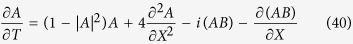



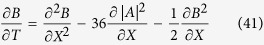


The above equations accept trivial solutions: 
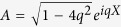
 and *B* = 0, which indicates a band of possible wavenumbers *q* in the close vicinity of 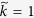. In order to study the dynamical stability of these solutions, we define *A* = (*A*_0_ + *r*)*e*^*i*(*qX*+*ϕ*)^ and *B* = *ψ* where *r*, *ϕ*, *ψ* are small real quantities of *X* and *T* compared to *A*_0_. The linearization of equations 40 and 41 gives:


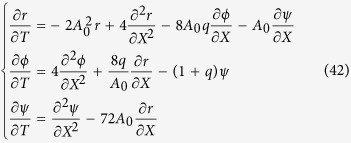


In the long wavelength limit, expanding *r*, *ϕ*, *ψ* as *e*^*ηt*+*ipx*^ (*p* being small) and solving the above system, one finally obtains:


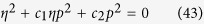


with *c*_1_ = (41 + 144*q* − 52*q*^2^)/(1 − 4*q*^2^) and *c*_2_ = −288*q*(*q* + 1). The two roots for the growth rate *η* are then


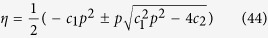


When *c*_2_ < 0, *q* > 0, equation (43) accepts two real solutions of opposite signs, giving a mode with exponential growth. Then, this domain *q* > 0 is forbidden. If *q* < 0 and *c*_2_ > 0, two complex roots exit and the stability of the front is thus given by the sign of *c*_1_ which has two roots: 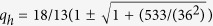, one being negative *q*_*h*_ ~ −0.260. In the interval of negative *q* between *q*_*h*_ and 0, *c*_1_ is positive, so static oscillatory modes exist around 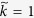. This stability stops at *q*_*h*_ but for this particular value, one reaches a Hopf bifurcation with a time dependent oscillation with frequency 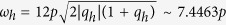.

## Additional Information

**How to cite this article**: Ben Amar, M. and Bianca, C. Onset of nonlinearity in a stochastic model for auto-chemotactic advancing epithelia. *Sci. Rep.* **6**, 33849; doi: 10.1038/srep33849 (2016).
